# Far and wide: Exploring provider utilization of remote service provision for genome‐wide sequencing in Canada

**DOI:** 10.1002/mgg3.1784

**Published:** 2021-09-17

**Authors:** Emily A. Enns, Tasha Wainstein, Nick Dragojlovic, Nicola Kopac, GenCOUNSEL Study, Larry D. Lynd, Alison M. Elliott

**Affiliations:** ^1^ Department of Human Genetics McGill University Montreal Quebec Canada; ^2^ Department of Medical Genetics University of British Columbia Vancouver British Columbia Canada; ^3^ Collaboration for Outcomes Research and Evaluation Faculty of Pharmaceutical Sciences University of British Columbia Vancouver British Columbia Canada; ^4^ Centre for Health Evaluation and Outcomes Sciences Providence Health Research Institute Vancouver British Columbia Canada; ^5^ BC Children’s Hospital Research Institute Vancouver British Columbia Canada; ^6^ Women’s Health Research Institute Vancouver British Columbia Canada

**Keywords:** access, genetic counseling, genome‐wide sequencing, remote service provision, telehealth, telemedicine

## Abstract

**Background:**

In Canada, funding for genome‐wide sequencing (GWS; exome and whole genome) is provincially regulated. We characterized the uptake of GWS by genetics health professionals (GHPs) across Canada and describe how they use remote technologies for patient access to GWS and genomic counseling.

**Methods:**

We distributed a survey to 574 Canadian GHPs addressing: GWS use, remote technologies (e.g., telephone, videoconferencing) for GWS and provider opinions regarding these technologies. Data were summarized using descriptive statistics. Associations between variables were evaluated using Chi‐square and Fisher's Exact tests for categorical data, and t‐tests or Mann–Whitney U tests for continuous data.

**Results:**

Of 116 GHPs, 50% reported using GWS in the last year and 57% of GWS users reported using remote technologies. Clinical geneticists who did not use GWS reported lack of provincial funding as the principal reason. Remote technologies were most commonly used for informed consent and results, and rarely used for initial consultations. Average wait times for a GWS appointment were shorter for remote appointments (mean 44.2 (SD 40.2) weeks) than for in‐person (mean 58.2 (SD 42.9), *p* = 0.036).

**Conclusion:**

The use of GWS varied across Canada, professional designation, and discipline. Funding remains a barrier to GWS access. Remote technologies increase patient access with reduced wait times.

## Graphical abstract

Responses regarding genome‐wide sequencing use across Canada.

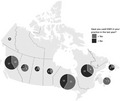

## 1 INTRODUCTION

Genome‐wide sequencing (GWS; exome and whole genome) is increasingly becoming a timely and resource‐efficient choice of genetic test, that is well suited for rare disease (Sawyer et al., 2016; Wright et al., 2018) and is now recommended as an early investigation in a patient's diagnostic odyssey (Srivastava et al., 2019). Canadian, American, and European guidelines recommend genetic counseling for all families considering GWS (Elliott & Friedman, 2018).

In Canada, the concentration of genetics clinics in large metropolitan centers requires many patients in remote and rural areas to travel far and wide to receive these services in‐person, placing a burden on families in the form of time away from work and home, the cost of travel and accommodations, and the risk of travel during inclement weather (Sevean et al., 2009). Our group has recently shown that travel costs and caregiver productivity loss associated with attending diagnosis‐related appointments for children with rare genetic disorders in British Columbia is approximately $1907/family/year (Dragojlovic et al., 2020). Since these factors can obstruct access to genetics services, some genetics health professionals (GHPs; i.e., geneticists, genetic counselors, etc.) offer alternative service delivery methods. In this study, we use the term “remote genetics service provision” (RGSP) to describe all strategies that are not “in‐person” (e.g., telephone, commercial videoconferencing software (e.g., Skype, Zoom), provincial telehealth programs (which involves patients travelling to a hospital or clinical unit near their place of residence), and online tools such as decision aids and webinars.

Studies have examined the use of RGSP within Canada, for indications such as prenatal counseling (Elliott et al., 2012), hereditary cancer (D’Agincourt‐Canning et al., 2008) and Huntington disease predictive testing (Hawkins et al., 2013a, 2013b). However, RGSP adoption for GWS has not been evaluated as clinical GWS is not available in all provinces. We have recently shown cost efficiencies and a delay to complete trio sample accrual with telehealth in a translational GWS pediatric research study in British Columbia (Elliott et al., 2021). To facilitate access to GWS, it is important to establish uptake and provider opinions of RGSP to identify the barriers to adoption. This study characterizes the uptake and barriers to GWS among Canadian GHPs and how Canadian GHPs use RGSP for GWS. This study is part of the GenCOUNSEL Study: “Optimization of genetic counselling for the clinical implementation of genome‐wide sequencing.”

## 2 METHODS

### 2.1 Survey development

We designed and administered a national survey to Canadian GHPs to assess the use of GWS in the last year and their experience with remote service provision for these cases. A questionnaire was developed in consultation with geneticists, genetic counselors, and researchers affiliated with the GenCOUNSEL study and McGill University Health Centre. The survey consisted primarily of closed‐ended questions as well as three open‐ended prompts for written comments. The questionnaire was divided into four categories: participant demographics, GWS use, RGSP for GWS (including length of appointments and wait times to appointments [as compared to in‐person appointments]), and, provider opinions regarding benefits and limitations of RGSP use in GWS. Participants who did not use GWS and/or RGSP had a shorter overall survey. When measuring opinions, participants were asked to rate their agreement with statements regarding certain benefits and limitations to RGSP for GWS using a 5‐point Likert scale, ranging from strongly disagree to strongly agree. For each statement, “agree” and “strongly agree” responses were added to derive percentage agreement, while “disagree” and “strongly disagree” were added to derive percentage disagreement (see supplementary material for questionnaire).

### 2.2 Study distribution

A letter of contact with a link to the online survey was distributed to practicing Canadian GHPs through email distribution lists from the Canadian Association of Genetic Counsellors (CAGC) and the Canadian College of Medical Geneticists (CCMG). The survey was distributed to active, eligible members only. The survey invitation was distributed to 309 full CAGC members and 265 CCMG members. Data were collected and stored in a secure REDCap database (Harris et al., 2009) at the BC Children's Hospital Research Institute. The survey was open for 4 months (September to December 2019).

### 2.3 Data analysis

Data were summarized using descriptive statistics. The statistical significance of associations between variables were evaluated using Chi‐square and Fisher's Exact tests for categorical data, and t‐tests or Mann–Whitney U tests for continuous data, depending on the distributions. Provinces and territories with a small number of respondents were combined together into regions to provide sufficient sample size for comparisons. Specifically, Yukon Territory + British Columbia; prairie provinces (Alberta, + Saskatchewan, + Manitoba); Atlantic provinces (Newfoundland + Nova Scotia) (no responses were received from remaining provinces or territories). To add context to our findings, open‐ended written responses were elicited from providers who reported using RGSP for GWS. These responses were coded and allocated into themes (Braun & Clarke, 2006). Coding and theme identification were conducted independently by two coders (EE and AME).

## 3 RESULTS

### 3.1 Survey response

In total, 127 responses were received, 11 of which were excluded: (three duplicates and eight incomplete). There were 116 valid responses from distribution to 574 members, for a total participation rate of 20.2%. A significantly greater proportion of CAGC members participated relative to CCMG members (29.8% (92 of 309) versus 9.1% (24 of 265); *χ* ^2^ *p* < .0005). Participant demographics are summarized in Table S1. The majority of participants were female, English‐speaking, worked at a public hospital or university‐affiliated medical center, in general genetics or cancer genetics as their primary area of practice, and lived in a large or very large city (population >300,000).

### 3.2 Use of GWS

Fifty percent (58/116) of respondents used GWS in the last year. Being a clinical geneticist, working in general genetics as a primary area of practice, and working at a university medical center were all significantly associated with GWS utilization (Table 1).

**TABLE 1:** Associations between provider characteristics and GWS use (N = 116 respondents).

Participants	Total N	Did not use GWS	Used GWS	Difference
		N (%)	N (%)	
Professional designation				
Genetic counsellors	91	50 (55)	41 (45)	Clinical geneticists are significantly more likely to use GWS than genetic counsellors (*χ* ^2^ = 10.486 (1) *p* = .001) (Note: Nurses & PhD Geneticists excluded)
Clinical geneticists	20	3 (15)	17 (85)	
Nurses	1	1 (100)	0 (0)	
PhD geneticists	4	4 (100)	0 (0)	
Primary area of practice				
General genetics	33	10 (30)	23 (70)	There is a significant association between area of practice and GWS utilization. (*χ* ^2^ = 13.57 (5 df) *p* = .016); (Fisher's Exact Test 13.869, *p* = .015) Those in general genetics appear to be more likely to use GWS than other specialties, while those in cancer genetics appear to be less likely.
Cancer	21	17 (81)	4 (19)	
Prenatal	12	6 (50)	6 (50)	
Pediatrics	8	4 (50)	4 (50)	
Research & Laboratory	16	9 (56)	7 (44)	
Other specialties	26	12 (46)	14 (54)	
Type of institution				
Public hospital/medical facility	53	27 (51)	26 (49)	Working at a university medical center is associated with a higher likelihood of GWS use (Fisher's exact test 12.854, *p* = .03).
University Medical Center	47	17 (36)	30 (64)	
Laboratory	5	4 (80)	1 (20)	
Other	11	10 (91)	1 (9)	
Years of practice				
Less than 6 years	40	21 (52.5)	19 (47.5)	Years of practice are not significantly associated with GWS utilization (*χ* ^2^ = 3.907 (2) *p*= 0.144)
6–15 years	40	23 (57.5)	17 (42.5)	
More than 15 years	34	12 (35)	22 (65)	
Province				
BC & Yukon	30	17 (57)	13 (43)	Province is not significantly associated with GWS utilization (*χ* ^2^ = 5.064 (4 df) *p* = .285)
Prairie provinces	15	7 (47)	8 (53)	
Ontario	36	15 (42)	21 (58)	
Québec	23	15 (65)	8 (35)	
Atlantic provinces	12	4 (33)	8 (67)	
Size of community				
Less than 1 million people	58	28 (48)	30 (52)	There were no significant differences between genetics professionals working in cities below 1 million inhabitants versus above 1 million inhabitants (*χ* ^2^ = 0.138 (1) *p* = .853)
More than 1 million people	58	30 (52)	28 (48)	

When the 58 respondents who did not use GWS in their practice over the last year were asked for their primary reason for not using GWS, 55% (n = 32) reported it was not clinically relevant to their practice, 38% (n = 22) reported that funding was not covered by the province, and 5% (n = 3) reported that no research opportunities with GWS were available (Table S2). The main reason for not using GWS varied by province (Fisher's Exact Test: *p* = .030) driven primarily by a difference between respondents from Ontario and British Columbia, the majority of whom (80% (12/15) and 71% (12/17), respectively) identified a lack of relevance to their practice as the main reason, whereas for those based in Quebec, only 27% (4/15) selected this reason. The majority of GHPs from Quebec (67%; 10/15) instead reported lack of provincial funding as the main reason.

Non‐users of GWS were represented across nearly all primary areas of practice (Table 1). GWS non‐users practicing primarily in general genetics and pediatrics reported that this was primarily due to a lack of provincial coverage (77.8% (7/9) and 100% (4/4)), respectively, rather than a lack of relevance to their practice. This differs significantly from GWS non‐users in cancer genetics (Fisher's Exact Test: *p* = .021), most of whom reported GWS was not relevant to their practice (65%; 11/17). Areas of practice where GWS was most relevant included general genetics (91%; 30/33), pediatrics (100%; 8/8), and metabolic disease (100%; 5/5); compared to the low rate of relevance in cancer genetics (43%; 9/21) *χ* = 21.2628; *p* < .00001).

### 3.3 Appointment characteristics of GWS and RGSP

Of the 58 respondents who used GWS in the last year, 57% (n = 33) reported that they used RGSP in at least one case involving GWS. There were no significant differences in RGSP uptake for GWS by profession, area of practice, or province. Respondents reported involvement in a median of 10 GWS cases in the past year (IQR = 5 to 23.75; n = 56). This translated to a median of 21 GWS appointments (IQR = 10 to 60), of which 29% (median 6 (IQR = 3 to 15)) conducted using RGSP. Respondents who reported using RGSP in the past year also reported more GWS cases overall per year (mean of 35.50 vs. 12.25; *p* = .04). While there was no significant difference between clinical geneticists and genetic counselors in the uptake of RGSP for GWS in the previous year, clinical geneticists were less likely to use RGSP in GWS appointments than GCs (12.7% of appointments vs. 37.4%, *p* = .02). Average GWS appointment length was 63 min (SD = 23), with an estimated average length of 38 min (SD = 17) for RGSP appointments and 89 min (SD = 81) for in‐person appointments (*p* < .0005). Average wait times for a GWS appointment were shorter for RGSP appointments (mean 44.2 weeks (SD 40.2), n = 26) than for in‐person appointments (mean 58.2 weeks (SD 42.9), *p* = .036).

### 3.4 Characteristics of RGSP for GWS

Among the respondents who used RGSP for GWS, the most common modalities were telephone and provincial telehealth programs (Table 2). One reported using email (entered under “other”). Of the three GHPs who reported using online tools, two used decision aids for pre‐test counseling, and one used written education material. All three who used online tools reported that it was to supplement, rather than replace, in‐person or remote counseling. No respondents reported using webinars or informed consent modules for GWS service delivery.

**TABLE 2:** Modalities of RGSP used by providers for GWS and genomic counselling (times per year each modality was used) as reported for the previous year (n = 32 respondents). Providers could select more than one modality.

Type of RGSP	Number of respondents reporting use (%)	Minimum number of uses per year	Maximum number of uses per year	Median number of uses per year
Telephone	24 (73)	1	45	5
Telehealth	23 (70)	1	50	3
Videoconferencing	1 (3)	4	4	4
Online tools	3 (9)	5	50	10
Other*	1 (3)	—	—	—

* Email.

Most RGSP users (88%; 28/33) reported using it for more than one part of the GWS service. In order of use, 92% of RGSP for GWS users reported using it for post‐test counseling and results (n = 30), 75% for informed consent (n = 24), 66% for pre‐test counseling (n = 21), and 47% for further follow‐up (n = 15). Only two respondents (6%) reported having used RGSP for the initial consultation/physical exam. One participant reported using RGSP for a pre‐appointment call to collect family history. The four respondents who used RGSP for only one component of GWS appointments, all reported using it for post‐test counseling and results.

We asked providers what types of facilities their patients accessed RGSP for GWS. Seventy‐two percent of providers reported that patients were seen at a hospital within the patient's community (n = 23), 53% at the patient's home (n = 17), 44% at a clinic/nursing station in the patient's community (n = 14), and 34% at a hospital or health unit outside of the patient's community (n = 11).

Reasons why respondents used RGSP instead of an in‐person session for GWS were assessed by multiple‐choice questions (Table S4). When asked to select the primary reason for offering RGSP for GWS cases, 63% selected travel.

### 3.5 Providers who did not use RGSP for GWS

Forty‐three percent (25/58) of GWS users did not use RGSP (Table 3) with 44% (11/25) reporting they did use RGSP in their practice for any indication.

**TABLE 3:** Reasons RGSP was not used for cases involving GWS, categorized according to professional designation. Participants could select more than one reason.

	Genetic counsellors N = 19 n (%)	Clinical geneticists N = 6 n (%)	Total N = 25 n (%)
1. Billing issues	0 (0)	0 (0)	0 (0)
2. Not standard practice	6 (32)	2 (33)	8 (32)
3. Patients are close by	11 (58)	2 (33)	13 (52)
4. Data security concerns	1 (5)	0 (0)	1 (4)
5. Provider access to technology	5 (26)	1 (17)	6 (24)
6. Patient access to technology	1 (5)	0 (0)	1 (4)
7. Technology is difficult, unreliable	0 (0)	0 (0)	0 (0)
8. Lack of interest	1 (5)	0 (0)	1 (4)
9. Other (not specified)	6 (32)	2 (33)	8 (32)

### 3.6 Provider opinions of benefits and limitations of using RGSP for GWS

Findings from Likert scale questions are shown in Figure S1a,b. Limitations of RGSP for GWS include increased difficulty with non‐verbal communication (72% in agreement) and psychosocial assessment (72% in agreement) compared to in‐person GWS consultations. Providers were unanimous in agreement that RGSP improves accessibility to GWS for remotely located patients and that RGSP for GWS is an efficient use of resources (e.g., time, travel cost).

Respondents were also given open response fields to write comments on the benefits, drawbacks, and any other considerations with respect to RGSP for GWS. Quotations were sorted into general themes (Tables S5–S8). Benefits identified included: patient appreciation (Q1‐3) improvement of patient access to GWS technology (Q4‐8), convenience and efficiency for the patient (Q9‐18), efficiency for the provider (Q19‐21), and reducing the burden of multistep workup (Q22‐24). A multistep workup was inherent to ordering GWS for some providers where GWS is not commonly accessible as a first‐line test:

*“At the institution where I work, offering GWS is a multi‐step process. We often order other baseline testing first. If we decide the patient should be offered GWS, we then discuss this with them to see if they are interested. If they are interested, we then apply to a governing body within the hospital (or sometimes at an outside lab/hospital depending on what province they live in) to determine if we get approval to order this testing (i.e., if the hospital will agree to cover the cost for this individual/family). If we do get approval, I then often follow‐up by phone or telehealth to discuss GWS in more detail and consent them to testing.” (Q22)*

A significant drawback to RGSP for GWS reported by respondents was the limited ability to perform the physical examination by RGSP (Q27‐35). The physical exam was emphasized as an essential component of the consultation for GWS cases.

“*For whole exome, we generally require one in‐person appointment for a physical exam, so it's unlikely that all appointments could be conducted via Telehealth*.” (Q27)

*“We do not provide RGSP for physician appointments at this time due to challenges with physical examination. It is only a service our clinic uses for genetic counselling and follow‐up appointments with physicians or genetic counsellors for result provision or relaying recommendations/management.”* (Q30)

Other drawbacks included logistical and technological challenges (Q36‐40; e.g., booking, sample collection), issues with communication and assessment of comprehension (Q41‐52), and increased difficulty with non‐verbal and psychosocial cues (Q53‐58).

Multiple providers spoke to the usefulness of RGSP for pre‐test counseling (Q68) and informed consent (Q63‐66).

*“This process is much easier for families and means that I can be giving them as much time as they need to discuss secondary findings, etc., without there being pressure for them to consent and sign a form the same day.”* (Q63)

RGSP was also useful for results and follow‐up (Q70‐75), depending on varying clinician and patient factors.

*“Results disclosure and other follow‐up is usually more amenable to RGSP if the patient would prefer not to travel to the clinic.”* (Q75)

One provider expressed that they envisioned RGSP to be used in the future to reduce wait times:

“*In my experience, RGSP is rarely or less frequently used for the initial consultation with physical assessment and genetic counselling*. *This part of the testing process is the greatest bottleneck to patients being seen in a reasonable timeframe, and I think RGSP could be very efficient in releasing this block, but would require significant clinical change and a shift in provider mindset*.” (Q33)

## 4 DISCUSSION

Our findings reflect the variability of GWS usage and RGSP for GWS across Canada and identifies benefits and drawbacks of using RGSP for GWS.

### 4.1 GWS access and usage

Half of the participating GHPs indicated they used GWS in their practice in the last year. The relevance of GWS to clinical practice varied by professional designation. More clinical geneticists used GWS than genetic counselors, and all geneticists who did not use it, indicated that this was due to a lack of provincial coverage, indicating variation in funding for GWS across Canada influences access to this technology for families.

GWS was used more frequently by clinicians in general, pediatric and metabolic genetics than in cancer genetics. The benefits of GWS over other testing strategies (multi‐gene panels, sequential testing) have been described to include potentially higher diagnostic yields (Clark et al., 2018; Lionel et al., 2018), a broader, unbiased testing strategy that can detect multiple genetic diseases in a single patient (Boycott & Innes, 2017), and reduced time to diagnosis and associated cost savings when used early in the patient's diagnostic trajectory (Tan et al., 2017). These benefits all appear to be of greatest value in general genetics rather than cancer genetics, which currently in Canada, is largely served by multi‐gene panels and familial testing.

### 4.2 Use of RGSP for GWS

Several studies have found a limiting factor in the adoption of RGSP for other indications is clinician comfort and acceptance (Otten et al., 2016; Wade et al., 2014), and that patient satisfaction with the service can be higher than that of the GHPs (D’Agincourt‐Canning et al., 2008; Gray et al., 2000). In our study, 43% of GHPs using GWS did not use RGSP for any part of the consultation and 56% of these indicated that they do not use RGSP in any case for other types of consultations. The most common reasons for RGSP non‐use included: patients resided near the treating hospital, RGSP was not standard practice, and that there were barriers to accessing the relevant technology, consistent with findings previously described (Otten et al., 2016).

The uptake of RGSP reported here (57% of GWS users, 29% of all appointments) for GWS was higher than the rates of 8% and 2% reported in the USA of GHPs for telephone and videoconferencing (Cohen et al., 2012), and 17% and 9% of GHPs reported in Europe (Otten et al., 2016). Motivations included reducing travel distance for patients, improving access to genetics services, and convenience and efficiency for both the patient and provider. Providers of RGSP for GWS appear to endure some of the drawbacks, such as difficulty with non‐verbal and psychosocial communication, in order to provide this service. Benefits of RGSP that are more specific to GWS include 1) reducing the travel burden of multi‐step workup and 2) allowing extra time to consider the relevant genomic counseling issues such as secondary findings and ethical implications (Ormond, 2013), rather than feeling pressured to consent on the same day. The themes generated by the open‐ended questions mirrored the issues interrogated on the Likert scale questions.

RGSP appointments in GWS cases were significantly shorter than in‐person appointments; this is different from other studies that showed that telehealth and in‐person appointments to be similar (Buchanan et al., 2015). This could be explained by the higher frequency of RGSP being used for results (i.e., calling out negative results by telephone) or informed consent, rather than the full initial assessment, which was rarely performed remotely.

The utility of RGSP may be different for GWS than for other types of genetic consultations for which RGSP has previously been studied in Canada, such as hereditary cancer (D’Agincourt‐Canning et al., 2008) and predictive testing protocols (Hawkins, Creighton, & Ho, 2013). In a pediatric GWS study, TH (through the provincial telehealth program in British Columbia) was offered to families for pre‐test counseling if the patient had been previously evaluated by a clinical geneticist, revealing cost efficiencies for families seen via TH but also a significant delay to complete sample accrual of the trio, resulting in potential delays for families who receive a diagnosis (Elliott et al., 2021). In the present study, while many respondents in our study used RGSP for pre‐and post‐test GWS counseling, only two providers reported using RGSP for the initial consultation and/or physical exam. This emerged as a strong theme among respondent comments: that GHPs required at least one in‐person appointment for a physical exam, often as the initial consultation (Table S6, Q27‐35). This represents a barrier to the completely remote provision of service for cases involving GWS. Some centers have reported success using telemedicine strategies, whereby geneticists conducted dysmorphology evaluation by real‐time video, although a genetic counselor or nurse was present to run the consultation and trained to take diagnostic measurements, and some dysmorphic features were missed (Hopper et al., 2011; Lea et al., 2005).

Previous studies have shown that remote service delivery improves patient access to genetics, including shorter wait times (Greenberg et al., 2020; Terry et al., 2019). In our study, the overall average wait‐time was lower for RGSP consults than in‐person consults. However, this benefit depended on the clinical approach, as some participants reported that patients who required physical examination prior to ordering GWS, wait times were not reduced.

### 4.3 Limitations

An ascertainment bias may be present if professionals who do not use GWS and RGSP were less likely to respond. However, the demographic proportions represented are comparable with those in Canada. Certain regions with few GHPs had small sample sizes and therefore responses from provinces in these regions were combined even though they have distinct provincial funding models.

The Canadian context of this study limits its generalizability to other countries with either approved public funding of GWS or privatized health care models. In these systems, there may be other factors that influence the use of both GWS and RGSP. Billing and reimbursement issues are cited as limiting factors to the use of RGSP in the USA (Cohen et al., 2016).

The rapid proliferation of COVID‐19 and associated physical distancing measures is serving as a catalyst for the use of remote service provision in novel ways and at new capacities in medical genetics services, as was predicted across all health sectors (Smith et al., 2020).

## 5 CONCLUSIONS AND PRACTICE IMPLICATIONS

This study demonstrated the uptake of GWS and RGSP identifying benefits and drawbacks. Travel distance for the patient was the primary reason for offering a GWS‐related consultation by RGSP. Wait times were less than for in‐person consultations, but the requirement of a physical examination necessitated in‐person appointments for many GHPs. This issue is even more relevant as COVID‐19 protocols limit the ability for GHPs to see patients in‐person. A move towards adoption of utilizing telehealth or videoconferencing for physical examination will remedy this issue. Provincial funding for clinical GWS remains a significant barrier to access in some jurisdictions.

## ETHICAL COMPLIANCE

Ethics approval for this study was obtained from the University of British Columbia Children's and Women's Hospitals Research Ethics Board. Informed consent was obtained from all participants.

## CONFLICT OF INTERESTS

The authors declared no competing conflicts of interest with respect to the research, authorship, and/or publication of this article.

## AUTHOR CONTRIBUTIONS

A.E. and E.E. contributed to conceptualization. E.E., T.W., and A.E. contributed to data curation. E.E., N.D., N.K., L.L., A.E., and T.W. contributed to formal analysis. A.E. and L.L contributed to funding acquisition. E.E., T.W., N.D., and A.E. contributed to methodology. T.W. and A.E. contributed to project administration and supervision. E.E. contributed to writing—original draft. E.E., A.E., L.L., N.D., T.W., and N.K. contributed to writing—review & editing.

## Supporting information

Supplementary Material

## References

[mgg31784-bib-0001] Boycott, K. M. , & Innes, A. M. (2017). When one diagnosis is not enough. New England Journal of Medicine, 376, 83–85. 10.1056/NEJMe1614384. 27959696

[mgg31784-bib-0002] Braun, V. , & Clarke, V. (2006). Using thematic analysis in psychology. Qualitative Research in Psychology, 3, 77–101. 10.1191/1478088706qp063oa

[mgg31784-bib-0003] Buchanan, A. H. , Datta, S. K. , Skinner, C. S. , Hollowell, G. P. , Beresford, H. F. , Freeland, T. , Rogers, B. , Boling, J. , Marcom, P. K. , & Adams, M. B (2015). Randomized trial of telegenetics vs. in‐person cancer genetic counseling: Cost, patient satisfaction and attendance. Journal of Genetic Counseling, 24, 961–970. 10.1007/s10897-015-9836-6 25833335 PMC4592683

[mgg31784-bib-0004] Clark, M. M. , Stark, Z. , Farnaes, L. , Tan, T. Y. , White, S. M. , Dimmock, D. , & Kingsmore, S. F. (2018). Meta‐analysis of the diagnostic and clinical utility of genome and exome sequencing and chromosomal microarray in children with suspected genetic diseases. NPJ Genomic Medicine, 3, 16. 10.1038/s41525-018-0053-8 30002876 PMC6037748

[mgg31784-bib-0005] Cohen, S. A. , Gustafson, S. L. , Marvin, M. L. , Riley, B. D. , Uhlmann, W. R. , Liebers, S. B. , & Rousseau, J. A. (2012). Report from the National Society of Genetic Counselors Service delivery model task force: a proposal to define models, components, and modes of referral. Journal of Genetic Counseling, 21, 645–651. 10.1007/s10897-012-9505-y 22566244

[mgg31784-bib-0006] Cohen, S. A. , Huziak, R. C. , Gustafson, S. , & Grubs, R. E. (2016). Analysis of advantages, limitations, and barriers of genetic counseling service delivery models. Journal of Genetic Counseling, 25, 1010–1018. 10.1007/s10897-016-9932-2 26762366

[mgg31784-bib-0007] D’Agincourt‐Canning, L. , McGillivray, B. , Panabaker, K. , Scott, J. , Pearn, A. , Ridge, Y. , & Portigal‐Todd, C. (2008). Evaluation of genetic counseling for hereditary cancer by videoconference in British Columbia. British Columbia Medical Journal, 50, 554–559.

[mgg31784-bib-0008] Dragojlovic, N. , van Karnebeek, C. D. M. , Ghani, A. , Genereaux, D. , Kim, E. , Birch, P. , Elliott, A. M. , Friedman, J. M. , & Lynd, L. D. (2020). The cost trajectory of the diagnostic care pathway for children with suspected genetic disorders. Genetics in Medicine, 22, 292–300. 10.1038/s41436-019-0635-6 31462755

[mgg31784-bib-0009] Elliott, A. M. , Dragojlovic, N. , Campbell, T. , Adam, S. , Souich, C. D. , Fryer, M. , Lehman, A. , Karnebeek, C. V. , Lynd, L. D. , & Friedman, J. M. (2021). Utilization of telehealth in paediatric genome‐wide sequencing: Health services implementation issues in the CAUSES Study. Journal of Telemedicine and Telecare, 1357633X2098273. 10.1177/1357633X20982737 33470133

[mgg31784-bib-0010] Elliott, A. M. , & Friedman, J. M. (2018). The importance of genetic counselling in genome‐wide sequencing. Nature Reviews Genetics, 19, 735–736. 10.1038/s41576-018-0057-3 30283055

[mgg31784-bib-0011] Elliott, A. M. , Mhanni, A. A. , Marles, S. L. , Greenberg, C. R. , Chudley, A. E. , Nyhof, G. C. , & Chodirker, B. N. (2012). Trends in telehealth versus on‐site clinical genetics appointments in Manitoba: A comparative study. Journal of Genetic Counseling, 21, 337–344. 10.1007/s10897-011-9406-5. 21997346

[mgg31784-bib-0012] Gray, J. , Brain, K. , Iredale, R. , Alderman, J. , France, E. , & Hughes, H. (2000). A pilot study of telegenetics. Journal of Telemedicine and Telecare, 6, 245–247. 10.1258/1357633001935329 11027129

[mgg31784-bib-0013] Greenberg, S. E. , Boothe, E. , Delaney, C. L. , Noss, R. , & Cohen, S. A. (2020). Genetic counseling service delivery models in the United States: Assessment of changes in use from 2010 to 2017. Journal of Genetic Counseling, 29, 1126–1141. 10.1002/jgc4.1265 32314856

[mgg31784-bib-0014] Harris, P. A. , Taylor, R. , Thielke, R. , Payne, J. , Gonzalez, N. , & Conde, J. G. (2009). Research electronic data capture (REDCap)—A metadata‐driven methodology and workflow process for providing translational research informatics support. Journal of Biomedical Informatics, 42, 377–381. 10.1016/j.jbi.2008.08.010 18929686 PMC2700030

[mgg31784-bib-0015] Hawkins, A. K. , Creighton, S. , & Hayden, M. R. (2013). When access is an issue: Exploring barriers to predictive testing for Huntington disease in British Columbia, Canada. European Journal of Human Genetics, 21, 148–153. 10.1038/ejhg.2012.147 22781094 PMC3548262

[mgg31784-bib-0016] Hawkins, A. K. , Creighton, S. , Ho, A. , McManus, B. , & Hayden, M. R. (2013). Providing predictive testing for Huntington disease via telehealth: results of a pilot study in British Columbia, Canada. Clinical Genetics, 84, 60–64. 23039041 10.1111/cge.12033

[mgg31784-bib-0017] Hopper, B. , Buckman, M. , & Edwards, M. (2011). Evaluation of satisfaction of parents with the use of videoconferencing for a pediatric genetic consultation. Twin Research and Human Genetics, 14, 343–346. 10.1375/twin.14.4.343 21787118

[mgg31784-bib-0018] Lea, D. H. , Johnson, J. L. , Ellingwood, S. , Allan, W. , Patel, A. , & Smith, R. (2005). Telegenetics in Maine: Successful clinical and educational service delivery model developed from a 3‐year pilot project. Genetics in Medicine, 7, 21–27. 10.1097/01.GIM.0000151150.20570.E7 15654224

[mgg31784-bib-0019] Lionel, A. C. , Costain, G. , Monfared, N. , Walker, S. , Reuter, M. S. , Hosseini, S. M. , Thiruvahindrapuram, B. , Merico, D. , Jobling, R. , Nalpathamkalam, T. , Pellecchia, G. , Sung, W. W. L. , Wang, Z. , Bikangaga, P. , Boelman, C. , Carter, M. T. , Cordeiro, D. , Cytrynbaum, C. , Dell, S. D. , … Marshall, C. R. (2018). Improved diagnostic yield compared with targeted gene sequencing panels suggests a role for whole‐genome sequencing as a first‐tier genetic test. Genetics in Medicine, 20, 435–443. 10.1038/gim.2017.119 28771251 PMC5895460

[mgg31784-bib-0020] Ormond, K. E. (2013). From genetic counseling to “genomic counseling”. Molecular Genetics & Genomic Medicine, 1, 189–193. 10.1002/mgg3.45 24498615 PMC3865587

[mgg31784-bib-0021] Otten, E. , Birnie, E. , Lucassen, A. M. , Ranchor, A. V. , & Van Langen, I. M. (2016). Telemedicine uptake among Genetics Professionals in Europe: Room for expansion. European Journal of Human Genetics, 24, 157–163. 10.1038/ejhg.2015.83 25898928 PMC4717205

[mgg31784-bib-0022] Sawyer, Sl , Hartley, T. , Dyment, Da , Beaulieu, Cl , Schwartzentruber, J. , Smith, A. , Bedford, Hm , Bernard, G. , Bernier, Fp , Brais, B. , Bulman, De , Warman Chardon, J. , Chitayat, D. , Deladoëy, J. , Fernandez, Ba , Frosk, P. , Geraghty, Mt , Gerull, B. , Gibson, W. , Gow, Rm , Graham, Ge , Green, Js , Heon, E. , Horvath, G. , Innes, Am , Jabado, N. , Kim, Rh , Koenekoop, Rk , Khan, A. , Lehmann, Oj , Mendoza‐Londono, R. , Michaud, Jl , Nikkel, Sm , Penney, Ls , Polychronakos, C. , Richer, J. , Rouleau, Ga , Samuels, Me , Siu, Vm , Suchowersky, O. , Tarnopolsky, Ma , Yoon, G. , Zahir, Fr , Majewski, J. , & Boycott, Km (2016). Utility of whole‐exome sequencing for those near the end of the diagnostic odyssey: time to address gaps in care. Clinical Genetics, 89, 275–284. 10.1111/cge.12654 26283276 PMC5053223

[mgg31784-bib-0023] Sevean, P. , Dampier, S. , Spadoni, M. , Strickland, S. , & Pilatzke, S. (2009). Patients and families experiences with video telehealth in rural/remote communities in Northern Canada. Journal of Clinical Nursing, 18, 2573–2579. 10.1111/j.1365-2702.2008.02427.x 19694885

[mgg31784-bib-0024] Smith, A. C. , Thomas, E. , Snoswell, C. L. , Haydon, H. , Mehrotra, A. , Clemensen, J. , & Caffery, L. J. (2020). Telehealth for global emergencies: Implications for coronavirus disease 2019 (COVID‐19). Journal of Telemedicine and Telecare, 26(5), 309–313. 10.1177/1357633X20916567 32196391 PMC7140977

[mgg31784-bib-0025] Srivastava, S. , Love‐Nichols, J. A. , Dies, K. A. , Ledbetter, D. H. , Martin, C. L. , Chung, W. K. , Firth, H. V. , Frazier, T. , Hansen, R. L. , Prock, L. , Brunner, H. , Hoang, N. , Scherer, S. W. , Sahin, M. , & Miller, D. T. (2019). Meta‐analysis and multidisciplinary consensus statement: exome sequencing is a first‐tier clinical diagnostic test for individuals with neurodevelopmental disorders. Genetics in Medicine, 21, 2413–2421. 31182824 10.1038/s41436-019-0554-6 PMC6831729

[mgg31784-bib-0026] Tan, T. Y. , Dillon, O. J. , Stark, Z. , Schofield, Deborah , Alam, Khurshid , Shrestha, Rupendra , Chong, Belinda , Phelan, Dean , Brett, Gemma R. , Creed, Emma , Jarmolowicz, Anna , Yap, Patrick , Walsh, Maie , Downie, Lilian , Amor, David J. , Savarirayan, Ravi , McGillivray, George , Yeung, Alison , Peters, Heidi , Robertson, Susan J. , Robinson, Aaron J. , Macciocca, Ivan , Sadedin, Simon , Bell, Katrina , Oshlack, Alicia , Georgeson, Peter , Thorne, Natalie , Gaff, Clara , & White, Susan M. (2017). Diagnostic impact and cost‐effectiveness of whole‐exome sequencing for ambulant children with suspected monogenic conditions. JAMA Pediatrics, 171, 855–862. 10.1001/jamapediatrics.2017.1755 28759686 PMC5710405

[mgg31784-bib-0027] Terry, A. B. , Wylie, A. , Raspa, M. , Vogel, Beth , Sanghavi, Kunal , Djurdjinovic, Luba , Caggana, Michele , & Bodurtha, Joann (2019). Clinical models of telehealth in genetics: A regional telegenetics landscape. Journal of Genetic Counseling, 28, 673–691. 10.1002/jgc4.1088 30825358

[mgg31784-bib-0028] Wade, V. A. , Eliott, J. A. , & Hiller, J. E. (2014). Clinician acceptance is the key factor for sustainable telehealth services. Qualitative Health Research, 24, 682–694. 10.1177/1049732314528809 24685708

[mgg31784-bib-0029] Wright, C. F. , FitzPatrick, D. R. , & Firth, H. V. (2018). Paediatric genomics: diagnosing rare disease in children. Nature Reviews Genetics, 19, 253–268. 10.1038/nrg.2017.116 29398702

