# Supplementary material for: Far and wide: Exploring provider utilization of remote service provision for genome‐wide sequencing in Canada
Source: Mol Genet Genomic Med. 2021 Sep 17;9(10):e1784. doi: 10.1002/mgg3.1784 (PMC8580085; doi:10.1002/mgg3.1784)
Supplement: Supplementary file 1 — Supplementary Material [file MGG3-9-e1784-s001.docx]

Supplementary Material

**Table S1.** Participant Demographics

|  | **Genetic counsellors and nurses (%)** | **Geneticists (%)** | **Total (%)** |
| --- | --- | --- | --- |
|  | ***N* = 92** | ***N =* 24** | ***N =* 116** |
| **Gender** |  |  |  |
| Male | 4 (4) | 11 (46) | 15 (13) |
| Female | 88 (96) | 13 (54) | 101 (87) |
| **Language** |  |  |  |
| English | 70 (76) | 16 (67) | 86 (74) |
| French | 4 (4) | 1 (4) | 5 (4) |
| Both | 17 (19) | 7 (29) | 24 (21) |
| **Professional designation** | | |  |
| Genetic counsellor | 91 (99) | 0 (0) | 91 (78) |
| Nurse | 1 (1) | 0 (0) | 1 (1) |
| Clinical geneticist (MD) | 0 (0) | 20 (83) | 20 (17) |
| PhD geneticist | 0 (0) | 4 (17) | 4 (3) |
| **Primary area of practice** |  |  |  |
| General genetics | 25 (27) | 8 (33) | 33 (28) |
| Cancer | 19 (21) | 2 (8) | 21 (18) |
| Prenatal | 9 (10) | 3 (13) | 12 (10) |
| Research | 8 (9) | 2 (8) | 10 (9) |
| Pediatrics | 8 (9) | 0 (0) | 8 (7) |
| Laboratory | 4 (4) | 2 (8) | 6 (5) |
| Metabolic disease | 2 (2) | 3 (13) | 5 (4) |
| Cardiology | 5 (5) | 0 (0) | 5 (4) |
| Genomic medicine | 3 (3) | 1 (4) | 4 (3) |
| Neurogenetics | 2 (2) | 1 (4) | 3 (3) |
| Infertility/ART/PIGD | 4 (4) | 0 (0) | 4 (4) |
| Newborn screening | 1 (1) | 0 (0) | 1 (1) |
| Other | 2 (2) | 2 (8) | 4 (3) |
| *Other, specified by participant: 2 “Ophthalmology,” 1 “Adult,” 1 "Combination of general, prenatal, pediatric, cancer, ocular"* | | | |
| **Type of institution** |  |  |  |
| Public hospital/medical center | 45 (49) | 8 (33) | 53 (46) |
| University medical center | 32 (35) | 15 (63) | 47 (41) |
| Private hospital/medical center | 7 (8) | 0 (0) | 7 (6) |
| Laboratory: non-commercial, academic | 3 (3) | 1 (4) | 4 (3) |
| University/Non-medical Center | 2 (2) | 0 (0) | 2 (2) |
| Laboratory: commercial, non-academic | 1 (1) | 0 (0) | 1 (1) |
| Other | 2 (2) | 0 (0) | 2 (2) |
| *Other, specified by participant= 1 "private telehealth GC company", 1 "newborn screening laboratory"* | | | |
| **Province/Territory** |  |  |  |
| British Columbia & Yukon | 25 (27) | 5 (21) | 30 (26) |
| Prairie Provinces  *(Alberta, Saskatchewan, Manitoba)* | 8 (8) | 7 (29) | 15 (13) |
| Ontario | 32 (35) | 4 (17) | 36 (31) |
| Quebec | 17 (19) | 6 (25) | 23 (20) |
| Atlantic Provinces  *(Nova Scotia, Newfoundland and Labrador)* | 10 (11) | 2 (8) | 12 (10) |
| *New Brunswick, PEI, NWT, Nunavut* | 0 (0) | 0 (0) | 0 (0) |
| **Size of city (population)** |  |  |  |
| Town (< 100,000) | 2 (2) | 0 (0) | 2 (2) |
| City (100,000 - 300,000) | 19 (21) | 3 (13) | 22 (19) |
| Large city (300,000 - 1 million) | 25 (27) | 8 (33) | 33 (28) |
| Very large city (>1 million) | 45 (49) | 13 (54) | 58 (50) |

**Table S2.** Provider reasons for not having used GWS in their practice in the last year.

|  | **Total**  **N** | **Not clinically relevant**  ***N (%)*** | **Not covered by province**  ***N (%)*** | **No research opportunities available**  ***N (%)*** | **Missing**  **N (%)** |
| --- | --- | --- | --- | --- | --- |
| **Total** | **58** | **32 (55)** | **22 (38)** | **3 (5)** | **1 (2)** |
| **Professional designation** | |  |  |  |  |
| Genetic counsellors | 50 | 27 (54) | 19 (38) | 3 (6) |  |
| Nurses | 1 | 1 (100) | 0 (0) | 0 (0) |  |
| Clinical geneticists | 3 | 0 (0) | 3 (100) | 0 (0) |  |
| PhD geneticists | 4 | 4 (100) | 0 (0) | 0 (0) |  |
| **Primary area of practice** | |  |  |  |  |
| General | 10 | 1 (10) | 7 (70) | 1 (10) | 1 (10) |
| Cancer | 17 | 11 (65) | 5 (29) | 1 (6) |  |
| Prenatal | 6 | 3 (50) | 3 (50) | 0 (0) |  |
| Research | 4 | 3 (75) | 0 (0) | 1 (25) |  |
| Pediatrics | 4 | 0 (0) | 4 (100) | 0 (0) |  |
| Laboratory | 5 | 4 (80) | 1 (20) | 0 (0) |  |
| Cardiology | 2 | 1 (50) | 1 (50) | 0 (0) |  |
| Genomic medicine | 2 | 2 (100) | 0 (0) | 0 (0) |  |
| Neurogenetics | 3 | 2 (67) | 1 (33) | 0 (0) |  |
| Fertility/ARD/PIGD | 4 | 4 (100) | 0 (0) | 0 (0) |  |
| Newborn screening | 1 | 1 (100) | 0 (0) | 0 (0) |  |
| **Province/Territory** | |  |  |  |  |
| BC | 17 | 12 (71) | 4 (23) | 1 (6) |  |
| Prairie Provinces  *(Alberta, Saskatchewan, Manitoba)* | 7 | 3 (43) | 3 (43) | 1 (14) |  |
| Ontario | 15 | 12 (80) | 3 (20) | 0 (0) |  |
| Quebec | 15 | 4 (27) | 10 (67) | 1 (6) |  |
| Atlantic Provinces  *(Nova Scotia, Newfoundland and Labrador)* | 4 | 1 (25) | 2 (50) | 0 (0) | 1 (25) |

**Table S3.** Characteristics of GWS use as reported by GHPs in their practice

1. **Number of cases that GHPs participated in last year, in which GWS was ordered**

|  | **Minimum** | **Maximum** | **Mean** | **StDev** | **Median** |
| --- | --- | --- | --- | --- | --- |
| Genetic counsellors  (N=40) | 1 | 100 | 15.7 | 19.04 | 10 |
| Clinical geneticists  (N=16) | 5 | 464 | 50.13 | 111.66 | 16 |
| Total  (N= 56) | 1 | 464 | 25.54 | 62.48 | 10 |

1. **Number of appointments scheduled with the patient for a typical case in which GWS is indicated**

|  | **1 appointment**  ***N (%)*** | **2 appointments**  ***N (%)*** | **3 appointments**  ***N (%)*** | **4 appointments**  ***N (%)*** |
| --- | --- | --- | --- | --- |
| Genetic counsellors  (N=40) | 6 (15) | 22 (55) | 10 (25) | 2 (5) |
| Clinical geneticists  (N=16) | 3 (19) | 8 (50) | 5 (31) | 0 (0) |
| Total  (N=56) | 9 (16) | 30 (53) | 15 (27) | 2 (4) |

1. **Duration of the average appointment**

|  | **<30 minutes**  ***N (%)*** | **30min-1hr**  ***N (%)*** | **1hr – 2hrs**  ***N (%)*** | **>2hrs**  ***N (%)*** |
| --- | --- | --- | --- | --- |
| Genetic counsellors  (N=40) | 1 (2) | 25 (63) | 14 (35) | 0 (0) |
| Clinical geneticists  (N=16) | 0 (0) | 9 (56) | 7 (44) | 0 (0) |
| Total  (N=56) | 1 (2) | 34 (61) | 21 (37) | 0 (0) |

**Table S4.** Reasons that RGSP was used in lieu of an in-person session as selected by GHPs *N (%)*

|  | **Select All**  **N=32 (%)** | **Primary reason**  **N=32 (%)** |
| --- | --- | --- |
| Convenience/efficiency for provider | 14 (44) | 4 (13) |
| Distance for patient | 29 (91) | 20 (63) |
| No genetics service at patient site | 20 (63) | 0 (0) |
| Patient constraints | 19 (59) | 5 (16) |
| Multiple appointments required | 15 (47) | 1 (3) |
| Allocation of provider resources | 13 (41) | 2 (6) |
| Online decision aids available | 0 (0) | 0 (0) |
| Other | 3 (9) | 0 (0) |

**Figure S1a.** Number of GHPs in agreement with statements regarding potential barriers to and limitations of RGSP use for GWS cases. N = 32 respondents.


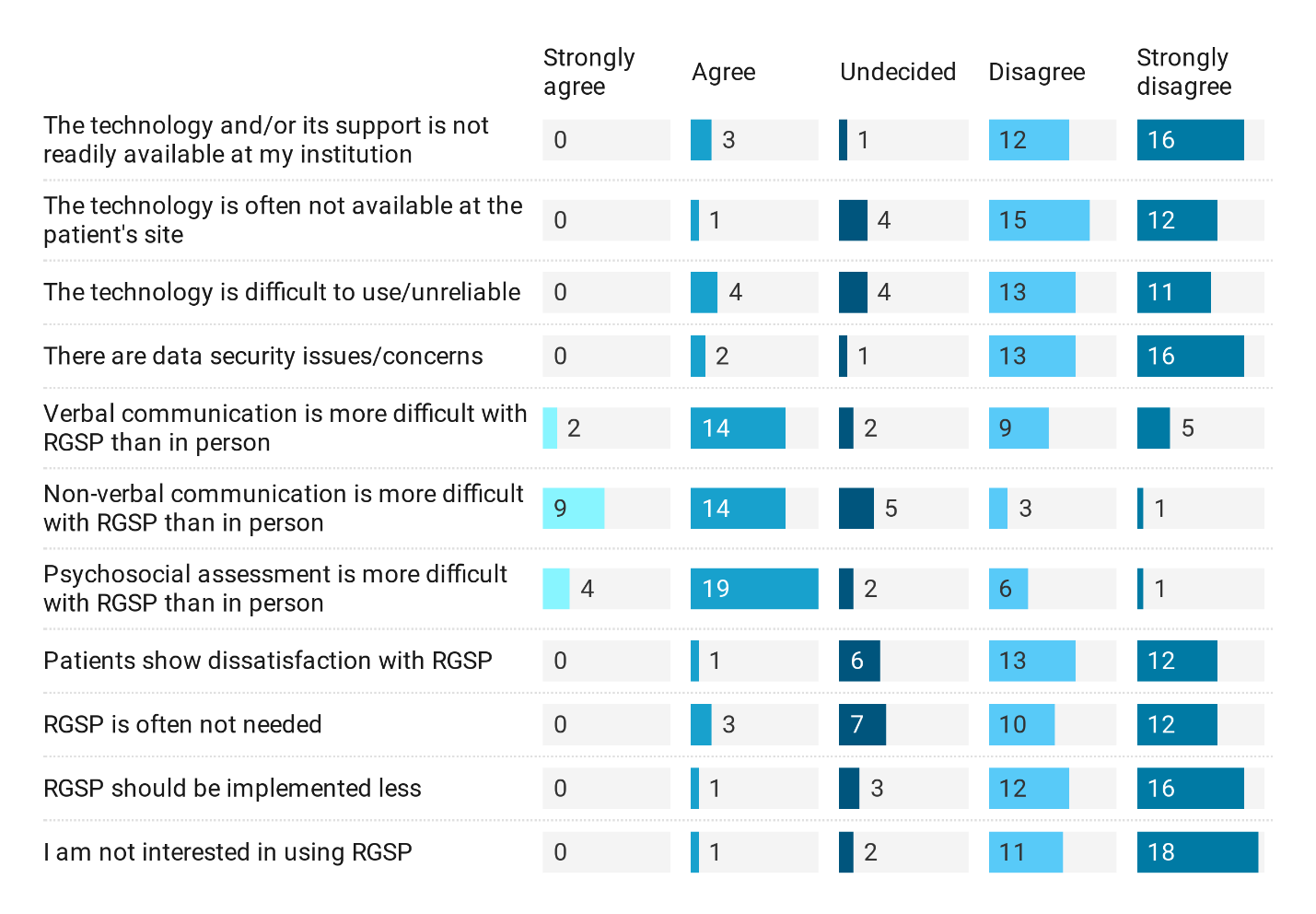


**Figure S1b.** Number of GHPs in agreement with statements regarding potential benefits of RGSP use for GWS cases. N = 32 respondents.


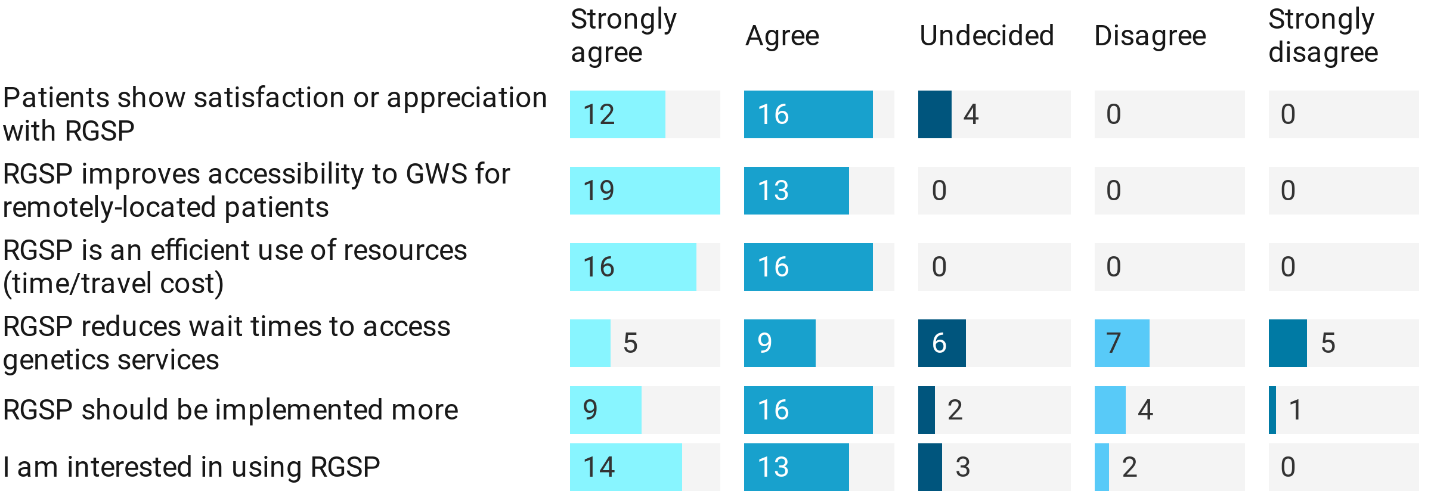


| **Table S5.** Respondent quotations regarding the *benefits* of using RGSP for GWS |
| --- |
| **1.1 Patient appreciation** |
| Q1. Patients commonly thank us for being able to access the service this way.  Q2. This improves patient satisfaction greatly.  Q3. Patient's/families who live a significant distance from the hospital are generally very happy to be offered telehealth or phone options. |
| **1.2 Improved patient access to service** |
| Q4. For some, the travel and time off work means they would not be able to access our service at all if we didn't have access to this technology.  Q5. […] increases access.  Q6. Big benefit that patients who live far from the tertiary care hospital don't have to travel to the big city for care.  Q7. Good tool to facilitate access to technology for remote location patients or patients with difficulties in traveling.  Q8. There are pros and cons to this type of service. However, there is a net benefit to the patient and there are some patients who wouldn't attend a Genetics appointment at all if they were not able to access our service this way. |
| **1.3 Convenience, efficiency, and cost savings for patient** |
| Q9. Patients love the convenience of not having to come in-person for an appointment  Q10. Reduce patient/family travel time and cost,  Q11. Convenience for the provider and the patient.  Q12. There are times when RGSP are very appropriate and effective, mainly creating cost efficiencies for the population I serve. I work in a remote community.  Q13. Reduces financial and travel burden for patients  Q14. We have patients that live anywhere from 2-15hrs away (driving) that fall into our catchment, so if we cannot coordinate an appointment for them while in town for other appointments it makes good sense to coordinate telehealth appointments to save the patient or family time/stress/money/etc.  Q15. The main benefit that you haven't mentioned in your survey is cost savings to the patient. This saves people a lot of time travelling and even more money. Less time taken off work where some people don't get paid if they aren't there, and the cost of cars, gas, food, hotel etc. So when you say cost savings, it isn’t a savings for me the physician or for the GC, it is a savings for the people. |
| Q16. Patients can use on own time, in own home  Q17. We are, currently, just using telephone counselling as a method of RGSP, mostly at the request of patients who prefer the convenience for time and geographical reasons.  Q18. Excellent way to ensure good pre and post-test counselling in remote patients without having to burden families with excessive travel. |
| **1.4 Efficiency for the provider** |
| Q19. I find that it also ensures that time is not wasted from the genetic counsellor perspective - patients that live farther away can encounter unexpected delays during travel which can result in showing up late for appointments or having to turn around and go home.  Q20. It is a more efficient use of my time (less time per patient). Patients who are especially involved/perceptive in their care often benefit immensely because they have done research previously and need less explanation. This is an efficient way of doing a psychosocial assessment and answering questions for these patients.  Q21. There are some instances where it makes a lot of sense and it is better use of everyone's time. |
| **1.5 Reduces burden of multi-step workup for GWS** |
| Q22. At the institution where I work, offering GWS is a multi-step process. We often order other baseline testing first. If we decide the patient should be offered GWS, we then discuss is with them to see if they are interested. If they are interested, we then apply to a governing body within the hospital (or sometimes at an outside lab/hospital depending on what province they live in) to determine if we get approval to order this testing (i.e. if the hospital will agree to cover the cost for this individual/family). If we do get approval, I then often follow-up by phone or telehealth to discuss GWS in more detail and consent them to testing. There will often be paperwork passed back and forth by email during this time as well as many labs want a signed consent form for GWS.  Q23. Facilitates access once primary assessment has been made, by preventing repeat visits  Q24. I use rgsp for patients I have not personally discussed gws with during md consultation |
| **1.6 Other benefits** |
| Q25. In the case of offering trio exome may be easier for both parents to be in attendance locally versus traveling to appointment  Q26. Potentially allows for more support individuals to be able to attend session with patient |

| **Table S6.** Respondent quotations regarding the *limitations/drawbacks* of RGSP for GWS |
| --- |
| **2.1 RGSP not useful for initial assessment/physical exam** |
| Q27. For whole exome, we generally require one in-person appointment for a physical exam, so it's unlikely that all appointments could be conducted via Telehealth.  Q28. Primary assessment (particularly in context of need for a physical examination) is very difficult to conduct by Telehealth and impossible by phone  Q29. Unable to examine patient.  Q30. We do not provide RGSP for physician appointments at this time due to challenges with physical examination. It is only a service our clinic uses for genetic counselling and follow-up appointments with physicians or genetic counsellors for result provision or relaying recommendations/management.  Q31. Main issue is deep phenotyping remains central to making WGS actually work. In UK 100,000 genomes restructure of labs so that there was no longer an in-person MDT to discuss cases and data ended up with multiple mis-diagnoses.  Q32. Does not have my favor for "first contact", when the therapeutic relationship is established |
| Q33. In my experience, RGSP is rarely or less frequently used for the initial consultation with physical assessment and genetic counselling. This part of the testing process is the greatest bottleneck to patients being seen in a reasonable timeframe, and I think RGSP could be very efficient and releasing this block, but would require significant clinical change and a shift in provider mindset.  Q34. …Although it cannot replace a physical exam, it is very helpful for all follow up services.  Q35. In our clinic, we almost never have initial assessments via RGSP; rather, we would see the patient in-person for their first consultation (permitting a physical exam to be completed and full counselling to be provided), and follow-up appointments for patient consent and results disclosure may be done via RGSP if the patient strongly prefers it or if travelling to the clinic would be an undue burden. |
| **2.2 Logistical and technological challenges** |
| Q36. Drawbacks are that it takes for resources in our clinic to book/coordinate. But the thought of having all of these people travel to see us in person, often for a 5 hour drive or longer, just doesn't sit well with me. We have the telehealth machines in our clinic space so access isn’t a problem  Q37. Technological difficulties can arise (sound, video not working) and it does take extra admin time to get organized.  Q38. One drawback of RGSP at my institution is the limited access to video communication and online visuals.  Q39. Frequent technical difficulties, lack of availability at remote site  Q40. Can be trickier logistically (i.e. needing to mail/fax paperwork rather than fully review in-person at appointment which for GWS requisitions can be particularly helpful to have more real time review). |
| **2.3 Communication and patient comprehension issues (e.g. lack of visual aids)** |
| Q41. RGSP removes several key communication strategies (visual aids, drawing pictures, body language to give information and assess understanding).  Q42. Communication is more difficult using RGSP. It is more difficult to assess a client's understanding of the information provided.  Q43. Because the person is not directly in front of you, the communication and evaluation are more difficult. It is likely that this would be easier if some shared visual aids could be used, but I have no experience with this to know for certain.  Q44. Patients who are having trouble understanding the information, especially visual learners, have even more trouble with RGSP due to the added communication barriers.  Q45. Use of visual aids is more challenging. However, we do have a "document camera" that allows us to work through visual aids in real time, use a pen, etc.  Q46. For some patients the lack of visuals (due to phone being the only available mode of communication) makes RGSP seem less useful and effective.  Q47. It is more difficult to assess a client's understanding of the information provided.  Q48. Can't fully assess understanding over phone  Q49. It does limit the flow of normal conversation as there can be delays/lags which can result in one person interrupting or speaking over the other person. To avoid this mishap, the genetic counsellor needs to be aware and ensure there are sufficient pauses when asking questions, checking in or introducing a new topics.  Q50. If pt has question, has to contact me by phone or email instead of getting answer immediately  Q51. More difficult to explain complex genetics concepts remotely  Q52. It also compounds the difficulty of communicating with patients where there is a significant language barrier. |
| **2.4 Difficulty with nonverbal and psychosocial cues** |
| Q53. Picking up on psychosocial cues can be more challenging.  Q54. Can be more difficult to assess/address non-verbal cues and aspects of counselling depending on the case  Q55. Lack of non-verbal cues.  Q56. Harder to do psychosocial counselling,  Q57. More difficult to read non-verbal cues.  Q58. Of course it can be harder to establish rapport and assess responses over video vs. in person. |
| **2.5 No drawbacks** |
| Q59. In my experience it has worked great and there have been no major drawbacks  Q60. I don't see drawbacks.  Q61. To me, GWS or not GWS, telehealth should be used a lot more. |
| **2.6 Other drawbacks or limitations** |
| Q62. It can be difficult for larger groups of people (ie. families) |

| **Table S7.** Comments regarding components of the GWS consultation |
| --- |
| - 1. **Initial consultation/physical exam** |
| See section 2.1 of Table S6 |
| **3.2 Informed consent** |
| Q63. This process is much easier for families and means that I can be giving them as much time as they need to discuss secondary findings, etc, without there being pressure for them to consent and sign a form the same day.  Q64. Once approval for testing is received I can quickly arrange a Telehealth to go over all of the consenting rather than waiting for an in person appointment.  Q65. RGSP helps with consent  Q66. Patients can take time in making informed decision |
| **3.3 Pre-test counselling** |
| Q68. I find it is particularly good for pretest counselling in our current system. We generally will not be seeing a patient specifically for GWS but it will come up in the process of our investigation that it is indicated. |
| **3.4 Results/Follow-up** |
| Q70. We use RGSP for giving results for everything when needed.  Q71. It's fine for results sharing or follow up. For initial assessment patient has to be assessed anyway so counselling and consenting can be done as part of the original assessment.  Q72. […] follow up is generally by telephone or telehealth depending on the outcome of the result.  Q73. These research GWS patients have a lot of questions, so numerous follow-up telephone counselling sessions have been done as well. I feel, however, that these follow-up calls would have been necessary even if the result session had been done in person.  Q74. I use RGSP for arranging testing, results, and follow up.  Q75. Results disclosure and other follow-up is usually more amenable to RGSP if the patient would prefer not to travel to the clinic. |

| **Table S8.** Other comments |
| --- |
| **4.1 Mixed reviews: Impact of RGSP on wait times** |
| Q76. It probably does lessen the wait time a bit, as we work these sessions in outside our organized clinic times.  Q77. RGSP does not typically reduce the wait time for the initial appointment. We have a wait time of around 18 months for a general patient that is non-urgent.  Q78. No one goes straight from referral to RGSP. They need an assessment (current non urgent cases wait 2 years) to decide if GWS is suitable. If it is suitable and counselling can't be done that day it can either be in person on RGSP. There the wait time depends more on external factors (funding decisions. client availability) |
| **4.2 Mixed reviews: No-show rates** |
| Q79. Patients also miss appointments more frequently.  Q80. Difficulty knowing whether a patient is a no show or just late to attend videoconference leading to time lost.  Q81. In general there are high no show rates for remote service appointments but in my experience at least this has not been an issue for those pertain to genome sequencing. Not sure why although I suspect families may be more motivated in these circumstances.  Q82. In general there are high no-show rates for Telehealth which is the primary mechanism by which I provide RGSP however this does not seem to be as much of an issue in this area. My interpretation is that people are more motivated when it gets to the point of GWS.  Q83. RGSP is a fantastic option for any clinic where the patient population does not often live locally. Anecdotally, patients are much more likely to attend appointments when they do not have the burden of travel. |
| **4.3 Extra tools to supplement RGSP** |
| Q84. It would be valuable to have good tools such as: written info (for most patients), info available online (for some patients) and possibly some more interactive online info (not necessarily just decision aids). Follow up result letters are important.  Q85. However, we do have a "document camera" that allows us to work through visual aids in real time, use a pen, etc. |
| **4.4 Don’t see a difference between GWS and other types of counselling** |
| Q86. To me, GWS or not GWS, telehealth should be used a lot more. |

**Supplementary Material – Questionnaire**

All genetics professionals practicing in Canada are welcome to participate in this study.

The goal of this study is to assess the current Canada-wide usage of remote methods of providing genetics services for genome-wide sequencing (GWS; exome and whole-genome sequencing). Examples include pre- and/or post-test counselling. Both research and clinical GWS can be included.

Remote genetics service provision (**RGSP**): includes methods of genetics service delivery (genetic counselling) that are not in-person. Examples of RGSP include telephone, videoconferencing (e.g. Skype, Zoom, Facetime), telehealth (e.g. coordinated appointment through a health authority), and online tools such as education modules and decision aids.

The questionnaire will take approximately 10-15 minutes to complete. You may omit any questions that you are not comfortable answering, **including those that might risk individually identifying you.**

**Participant demographics**

1. What is your professional designation?
   1. Genetic counsellor
   2. Clinical geneticist (MD)
   3. PhD geneticist
   4. Nurse
   5. Other _____________
2. Are you:
   1. Female
   2. Male
   3. Other/Prefer not to answer
3. In what languages do you practice? Select all that apply.
   1. French
   2. English
   3. Other: ___________
4. How many years have you been practicing in genetics since the completion of your training?
   1. Sliding scale: 1 – 100
5. What is your current **primary** area of practice as of the last year? Select one.
   1. General Genetics
   2. Pediatrics
   3. Prenatal
   4. Cancer
   5. Cardiology
   6. Neurogenetics
   7. Psychiatric
   8. Preconception/Reproductive Screening
   9. Metabolic Disease
   10. Laboratory
   11. Research
   12. Genomic Medicine
   13. Pharmacogenetics
   14. Public Health
   15. Newborn screening
   16. Infertility, ART/IVF, PGD
   17. Other ____________________
6. In what type of institution do you currently **primarily** practice?
   1. Public Hospital/Medical Facility
   2. University Medical Center
   3. Diagnostic Laboratory – Commercial, Non-academic
   4. Diagnostic Laboratory – Non-commercial, Academic
   5. Private Hospital/Medical Center
   6. University/Non-medical Center
   7. Other ________________
7. In what province/territory do you currently practice? Select all that apply.
   1. Newfoundland and Labrador
   2. Prince Edward Island
   3. New Brunswick
   4. Nova Scotia
   5. Quebec
   6. Ontario
   7. Saskatchewan
   8. Manitoba
   9. Alberta
   10. British Columbia
   11. Nunavut
   12. Northwest Territories
   13. Yukon Territory
8. What is the size of the city in which you currently practice?
   1. Rural area/small town (<10,000 people)
   2. Town (10,000 – 100,000 people)
   3. City (100,000 – 300,000 people)
   4. Large city (300,000 – 1 million people)
   5. Very large city (>1 million people)

**Genome-wide sequencing (GWS)**

Genome-Wide Sequencing (GWS) includes **whole-exome** and **whole-genome** sequencing. Please include both research and clinical uses of GWS when answering these questions.

1. Have you used genome-wide sequencing (GWS) in your practice **in the last year**?
   1. Yes
   2. No

[If no: Question 10]

1. What are the reasons? Select all that apply.
   1. Not clinically relevant to my practice
   2. Not covered in my province/territory
   3. No research opportunities available

[If no: Thank you for your participation in this study. (End)]

[If yes: Continue]

1. In the past year, how often have you participated in cases in which GWS was ordered?
   1. Sliding scale: 1 – 100
2. In the average case in which GWS is indicated, how many appointments are usually scheduled with the patient?
   1. 1
   2. 2
   3. 3
   4. 4
   5. >5
3. How long is the average appointment?
   1. <30min
   2. 30min-1hr
   3. 1hr-2hrs
   4. >2hrs

**Remote genetics service provision (RGSP) and GWS**

These questions address the use of remote genetics service provision (**RGSP**) methods (e.g. telephone, videoconferencing, telehealth, online tools) in the context of cases that require genome-wide sequencing.

1. Have you used RGSP for patient contact in a case that involved GWS?
   1. Yes
   2. No
2. If no, what are the reasons? Select all that apply.
   1. Billing issues
   2. It is not standard practice at my institution
   3. Patients are geographically close by
   4. Issues with server or data security
   5. The technology and/or its support is not readily available at my institution
   6. The technology is often not available at the patient’s site
   7. The technology is difficult to use/unreliable
   8. I am not interested in using RGSP
   9. Other: ________________
3. Do you use RGSP for indications other than GWS (e.g. hereditary cancer genetic counselling?

a) Yes

b) No

[If no to question 14 : Thank you for your participation in this study. (End)]

[If yes: Continue]

1. What types of RGSP do you use for GWS cases? Select all that apply.
   1. Telephone
   2. Videoconferencing (e.g. Skype, Zoom, FaceTime)
   3. Provincial Telehealth program
   4. Online tools (e.g. decision aids, webinars, informed consent modules, educational material)
   5. Other: _________________________
2. Approximately how many times per year do you use each method of RGSP for GWS cases?
   1. Telephone: _____
   2. Videoconferencing (e.g. Skype, Zoom, FaceTime): ______
   3. Provincial/Regional Telehealth program: ______
   4. Online tools: ______
   5. Other: _________________________

[If selected “online tools” in Q17, go to Q20/21]

1. What type of online tools do you use?
   1. Decision aids
   2. Webinars
   3. Informed consent module
   4. Written educational material
   5. Other: ________
2. What do you use online tools for?
   - 1. Pre-test genetic counselling (e.g. decision aids)
     2. Informed consent
     3. General patient education (e.g. webinar)
     4. Other: ____________
3. Do you mostly use online tools *instead* of providing in-person/remote counselling or to *supplement* in-person/remote counselling?
4. Instead of in-person/remote counselling
5. To supplement in-person/remote counselling
6. Other: ____________

[If selected telephone, videoconferencing, or telehealth in Q 17, go to Q22]

1. About how long is the average telephone/videoconferencing/telehealth appointment?
2. <30 min
3. 30min-1hr
4. 1hr-2hrs
5. >2hrs
6. What are the reasons that you use RGSP in lieu of an in-person session for GWS? Check all that apply.
   1. Convenience and efficiency for the genetics health care provider
   2. Patient lives a long distance from the hospital
   3. No genetics services available at patient’s location
   4. Time/work/other constraints on patient’s ability to attend in-person
   5. Multiple appointments required for the case
   6. Allocation of genetics professional resources (e.g. time efficiency)
   7. Online decision aids available
   8. Other: ______________

1. What is the **primary** reason that you use RGSP in lieu of an in-person session for GWS? (Choose one)
   1. Convenience and efficiency for the genetics health care provider
   2. Patient lives a long distance from the hospital
   3. No genetics services available at patient’s location
   4. Time/work/other constraints on patient’s ability to attend in-person
   5. Multiple appointments required for the case
   6. Allocation of genetics professional resources (e.g. time efficiency)
   7. Online decision aids available
   8. Other: ______________
2. When using RGSP for GWS, in what types of facilities are patients counselled? Check all that apply.
   1. At their home
   2. A clinic/nursing station in the patient’s community
   3. A hospital in the patient’s community
   4. A hospital or health unit outside of the patient’s community
   5. Other: ________________
3. For what parts of the GWS consult have you used RGSP? Check all that apply.
   1. Initial consultation/physical examination
   2. Pre-test genetic counselling
   3. Informed consent
   4. Post-test genetic counselling (results, etc.)
   5. Further follow-up
   6. Other:________________

1. Estimate the average time (in weeks) a patient has to wait for an in-person GWS consultation at your clinic, beginning from the time of referral.
   1. Enter integer
2. Estimate the average time (in weeks) a patient has to wait for an RGSP consultation for GWS at your clinic, beginning from the time of referral.
   1. Enter integer
3. The following statements address potential barriers and limitations to using RGSP **for GWS cases**. To what extent do you agree with the following statements? (Likert Scale: Strongly agree, agree, undecided, disagree, strongly disagree)
   1. The technology and/or its support is not readily available at my institution
   2. The technology is often not available at the patient’s site
   3. The technology is difficult to use/unreliable
   4. There are data security issues/concerns
   5. Verbal communication is more difficult with RGSP than in person
   6. Non-verbal communication is more difficult with RGSP than in person
   7. Psychosocial assessment is more difficult with RGSP than in person
   8. Patients show dissatisfaction with RGSP
   9. RGSP is not often needed
   10. RGSP should be implemented less
   11. I am not interested in using RGSP
4. The following statements address potential benefits to using RGSP **for GWS cases**. To what extent do you agree with the following statements? (Likert Scale: Strongly agree, agree, undecided, disagree, strongly disagree)
   1. Patients show appreciation or satisfaction with RGSP
   2. RGSP improves accessibility to GWS for remotely-located patients
   3. RGSP is an efficient use of resources (time/travel cost)
   4. RGSP reduces wait times to access genetics services
   5. RGSP should be implemented more
   6. I am interested in using RGSP
5. Please comment on the benefits of RGSP for GWS in your experience:
   1. ______________________
   2. No additional comments
6. Please comment on the drawbacks of RGSP for GWS in your experience:
   1. _______________________
   2. No additional comments
7. Please provide any additional comments about the use of RGSP for GWS related genetic counselling
   1. ______________________
   2. No additional comments
